# The Enigma of Crohn’s Disease: A Case Report

**DOI:** 10.7759/cureus.55993

**Published:** 2024-03-11

**Authors:** Nabil Azmi, Warren Tiew Toot Chaw, Nurafdzillah Abdul Rahman, Sumayyah Zaihan, Wan Syahira Ellani Ahmad Kammal

**Affiliations:** 1 Department of Surgery, Faculty of Medicine, The National University of Malaysia, Kuala Lumpur, MYS; 2 Department of Pathology, Faculty of Medicine, The National University of Malaysia, Kuala Lumpur, MYS; 3 Department of Pathology, Faculty of Medicine and Health Sciences, Universiti Putra Malaysia, Kuala Lumpur, MYS

**Keywords:** colorectal, crohn's fistula, crohn's stricture, crohn’s disease (cd), inflammatory bowel syndrome

## Abstract

Crohn's disease (CD) is an inflammatory condition affecting the gastrointestinal tract, often leading to persistent inflammation in various parts, notably the terminal ileum and colon. Clinical manifestations of CD can become complex due to complications like strictures, fistulas, and perianal abscesses. The disease typically exhibits transmural inflammation and skip lesions, where unaffected areas alternate with affected ones. Treatment goals focus on achieving disease remission and preventing complications that may require surgical intervention. Surgery becomes crucial in managing complications such as Crohn's strictures and perforations. Here, we describe a challenging case involving a young woman who underwent surgery for abdominal abscess and with Crohn’s stricture and fistula.

## Introduction

Crohn’s Disease (CD) affects approximately 3 to 20 individuals per 100,000 globally each year [1]. It is characterized by chronic inflammation of the digestive tract, which extends through its full thickness (transmural) and is marked by granulomas. Typically, the disease manifests in early adulthood, presenting symptoms such as recurring abdominal pain, diarrhea, unintentional weight loss, and occasionally extraintestinal symptoms like rash and joint pain. The clinical presentation varies depending on the location and severity of the disease. While several causes of CD have been proposed, the most widely accepted theory implicates a dysregulated immune response resulting from the interaction of gut microbiota, genetic predisposition, and environmental factors [2]. CD often presents with a range of complications, such as obstruction, strictures, abscesses, fistulas, and gastrointestinal bleeding. Despite advancements in drug therapy, including biological treatments, surgical intervention with bowel resection remains crucial in managing these patients and can be life-saving [3]. Here, we present a challenging case of a patient with CD complicated by colo-colic and entero-enteric fistulas, as well as bowel stricture, who was successfully treated with surgery after medical therapy proved ineffective.

## Case presentation

**Figure 1:**
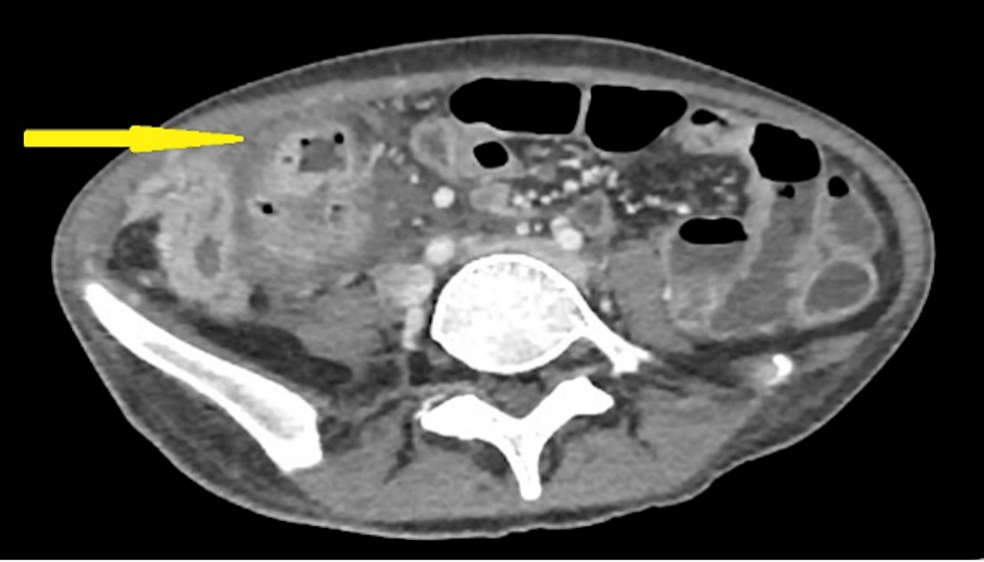
CT scan (Axial cut) showing a heterogenous enhancing collection (yellow arrow) at the right paracolic gutter

**Figure 2:**
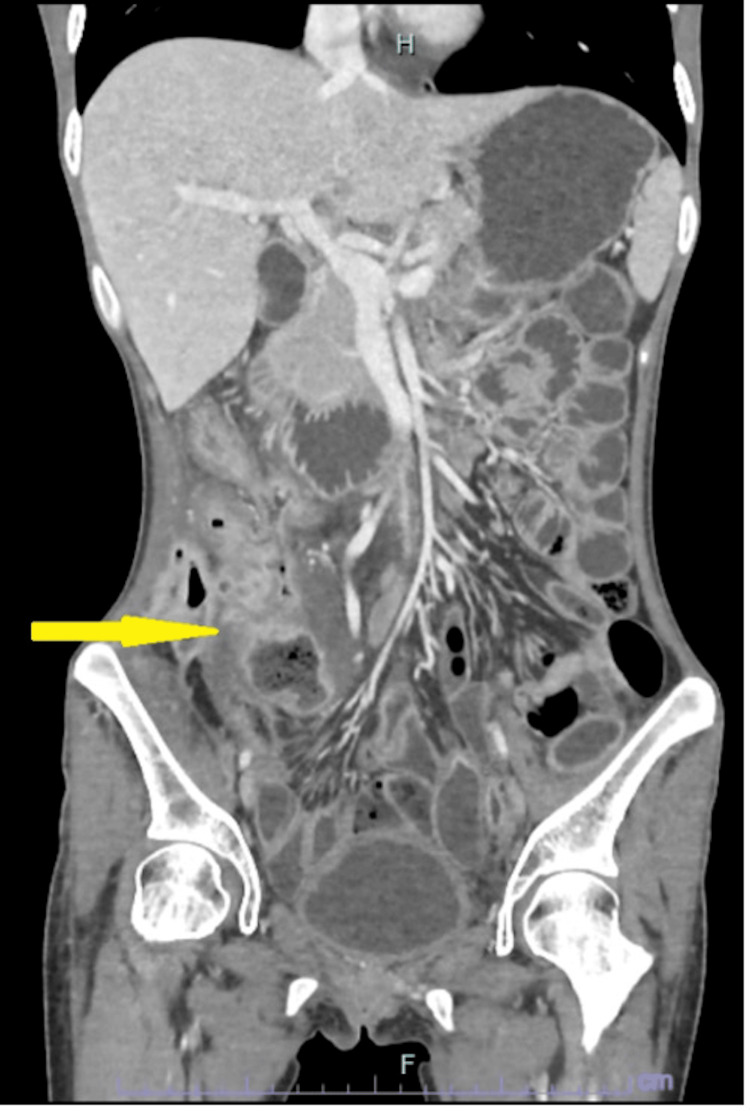
CT scan (coronal view) showing a heterogenous collection (yellow arrow) at the right paracolic gutter with small air locules within.

A 22-year-old woman diagnosed with ileocecal CD, who had been treated with immunosuppressive medication Azathioprine and biologics named Vedolizumab, presented to the emergency department after experiencing fever, lower abdominal pain, lethargy, and significant weight loss over the past week. Upon initial examination, she exhibited tenderness on the right side of her abdomen and was found to be febrile with a temperature of 38°C, but otherwise had normal blood pressure. Relevant blood tests including full blood count (FBC), renal profile (RP), liver function test (LFT), serum amylase, and C-reactive protein (CRP) were within normal ranges. Her calculated BMI indicated she was underweight, with a value of 18. A computed tomography (CT) scan of the abdomen and pelvis (Figure 1 and Figure 2) intraabdominal collection at the right paracolic gutter near the ascending colon and caecum with surrounding involvement of both large and small bowel.

**Figure 3:**
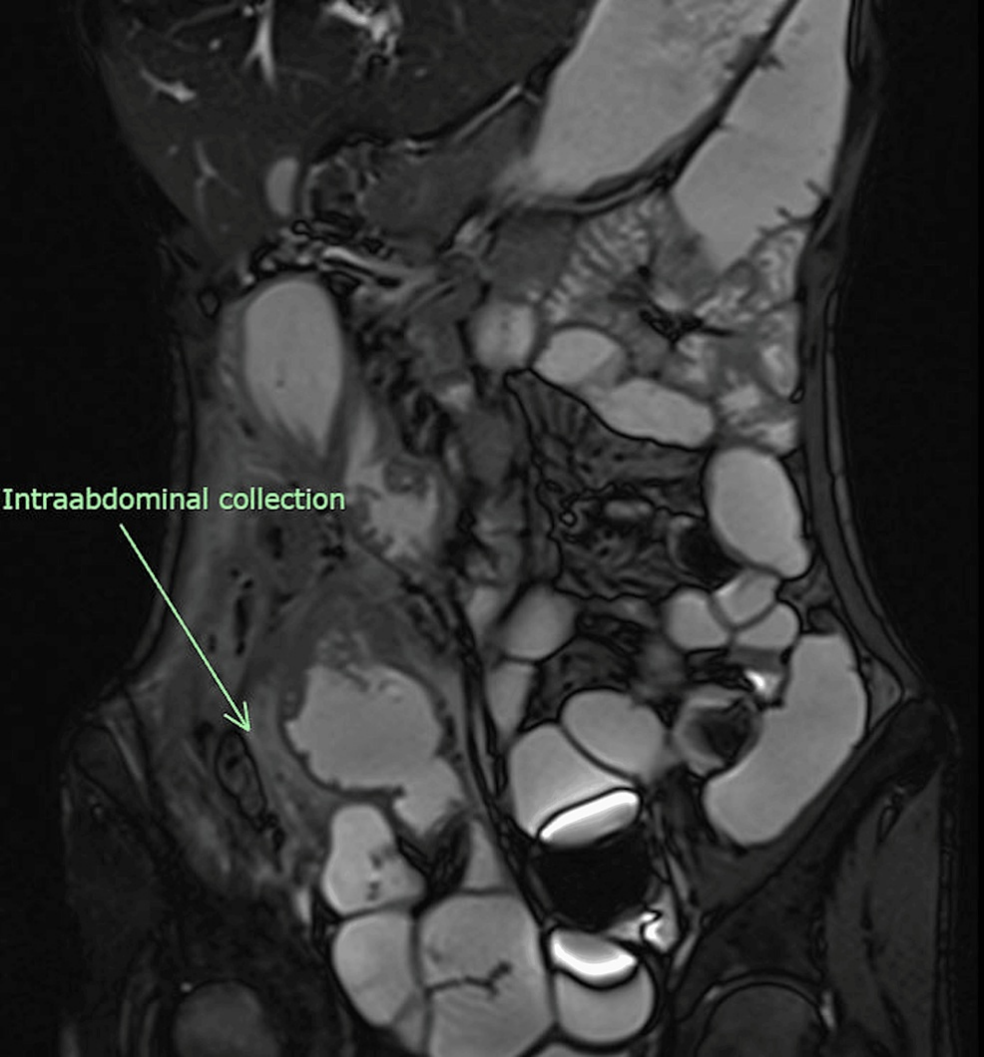
MRE in coronal view depicting intraabdominal collection and bowel inflammation

**Figure 4:**
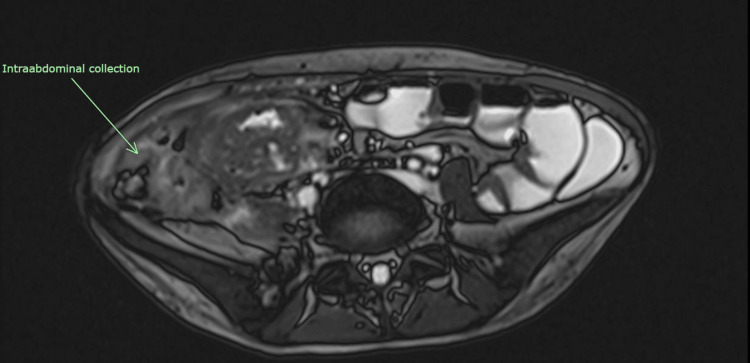
MRE in axial view depicting intraabdominal collection and inflamed bowel wall, the hallmark of Crohn's disease

The patient initially received antibiotic therapy for a total duration of 3 weeks along with percutaneous drainage to address the abscess. A colonoscopy was not performed during this admission as it would not only add value to management but would increase the risk of bowel perforation. However, despite these interventions, her sepsis persisted, and subsequent CT scans showed no improvement. Magnetic resonance enterography (MRE) was then conducted to assess the extent and activity of the disease. MRE is a valuable radiological assessment tool to assess disease severity in Crohn’s disease. Bowel wall inflammation is the hallmark of Crohn’s disease and, as in this case, was demonstrated clearly. Medical treatment with biologics had failed to achieve remission. Moreover, it provided an in-depth understanding of the anatomy of the fistulizing bowel and aided in the planning of surgery. Fistulizing disease (entero-colic) was observed in the right iliac fossa and mid-pelvic regions, along with interloop and right iliac fossa collections as depicted in Figures 3 and 4.

The case was reviewed in the hospital's multidisciplinary meeting, which included colorectal surgeons, gastroenterologists, interventional radiologists, and anesthesiologists. The consensus was to proceed with surgery following the optimization of the patient's condition and obtaining consent from both the patient and family members.

**Figure 5:**
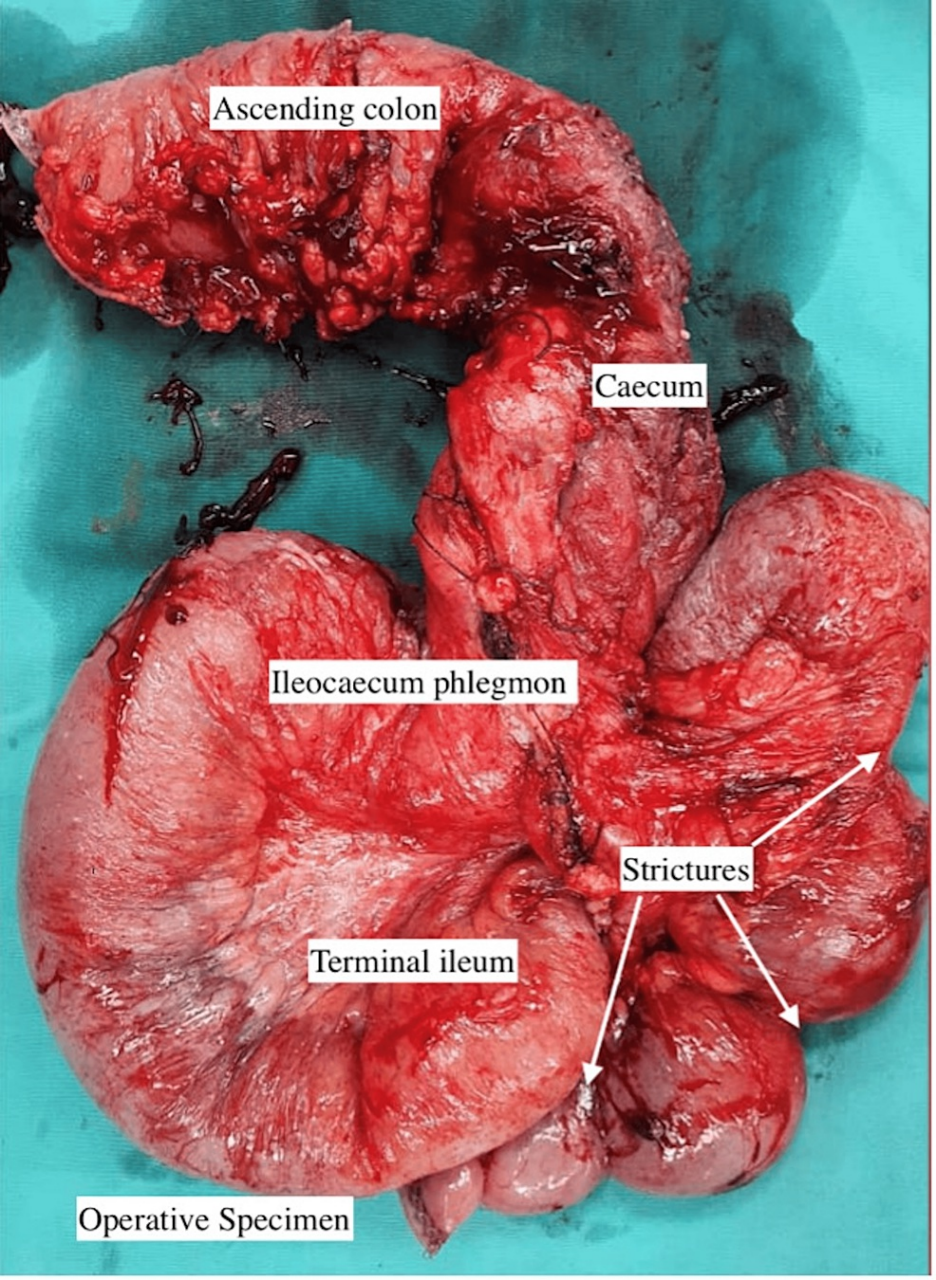
Thickened bowel with a creeping fat sign and multiple strictures can be observed in the resected specimen

Colorectal surgeons conducted an exploratory laparotomy and performed a right-sided hemicolectomy with a double-barrel stoma. The surgery lasted approximately five hours and resulted in minimal blood loss. Intraoperatively, the surgeons discovered a colo-colic fistula between the ascending colon and transverse colon, as well as an ileo-ileal fistula. Additionally, two areas of short stricture were identified in the ileum, accompanied by segmental dilatation, as depicted in Figure 5.

**Figure 6:**
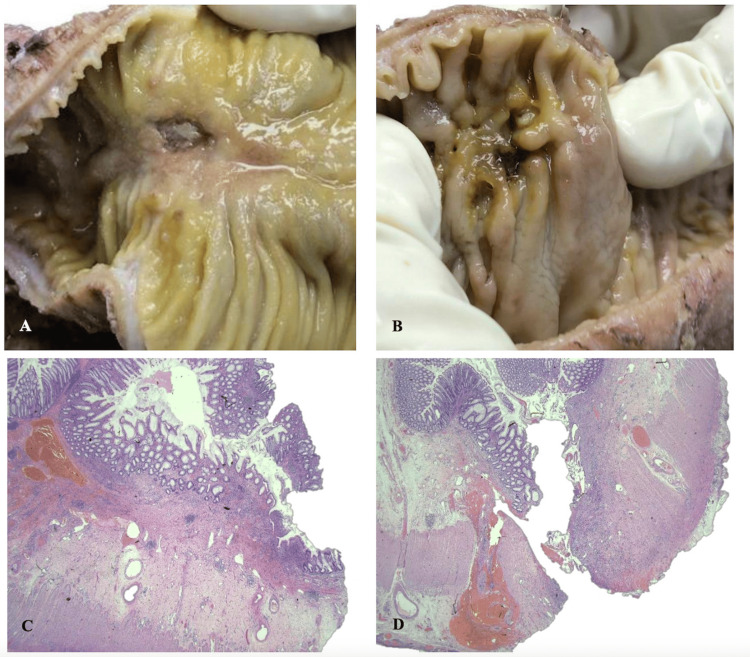
Macroscopic examination revealed A) deep fissuring ulcer within the small intestine and B) ulcers with fistula within the large intestine. C)Microscopically, there were multiple skipped ulcers separated by normal mucosa (H&E stain x20 magnification). D) Fistula site showing mucosal ulceration and muscularis propria disruption with heavy mixed inflammatory cells infiltrates and granulation tissue formation (H&E stain x20 magnification)

The specimen was sent for histopathological examination, which revealed chronic active ileitis and colitis. The examination also showed segmental ulcers, transmural inflammation, and fistula, as described in Figure 6.

The patient had a smooth post-surgery recovery and was discharged home well to continue outpatient clinic follow-up and medical therapy for CD.

## Discussion

CD is a chronic, remitting condition primarily caused by granulomatous transmural inflammation, often identifiable by macroscopic features such as skip lesions, ulceration, or cobblestone-like lesions within the gastrointestinal tract [4]. It typically follows a relapsing and remitting course, characterized by significant morbidity during flare-ups. Symptoms experienced by affected individuals can be debilitating. This includes chronic diarrhea, bleeding, and severe malnutrition. CD can affect any part of the gastrointestinal tract, from the mouth to the anus, often with multifocal involvement, and the small intestine, especially the terminal ileum, tends to be more affected [5]. Additionally, CD may manifest with extraintestinal symptoms such as skin or mouth lesions, arthralgia, eye irritation, cholelithiasis, nephrolithiasis, and other hepatobiliary disorders. It affects both men and women equally across all age groups, with susceptibility peaking in the second and third decades of life, and familial predisposition observed in some cases [6].

The management of CD primarily relies on medical therapy, which includes antimicrobial agents, 5-aminosalicylic acid (5-ASA) compounds like mesalazine, azathioprine, systemic corticosteroids, and anti-tumor necrosis factor (TNF) therapy such as Infliximab and Adalimumab. Successful long-term remission can be achieved in up to 35% of patients treated with multiple drug combinations [7]. However, surgery becomes necessary in cases of CD complicated by phlegmons, perforations, fistulas, abscesses, or intestinal partial or complete obstruction [8], as observed in this patient. Control of intra-abdominal sepsis mainly involves antimicrobial therapy, image-guided aspiration or drainage, surgical resection of the diseased bowel, and adequate nutritional support, which remain essential aspects of treatment. Conservative management with percutaneous image-guided catheter drainage is the preferred initial option if surgery carries significant morbidity. Successful percutaneous drainage may prevent surgery in up to 30% of patients , as reported by Torres J et al [8]. However, despite the effectiveness of percutaneous drainage and antibiotics in controlling sepsis, surgical resection may still be necessary, as the incidence of abscess recurrence following percutaneous drainage alone can be 6.5 times higher than that following percutaneous drainage combined with surgical resection [8]. Percutaneous drainage can be considered as a bridge to elective surgery, helping to optimize nutritional and medical conditions, and resulting in improved postoperative outcomes.

In cases where patients have optimal preoperative nutrition and favorable intraoperative conditions, such as adequate blood supply to the bowel and stable hemodynamics, a primary anastomosis is generally recommended. However, in situations where conditions are less favorable, including septic shock requiring inotropic support, blood acidosis, gross contamination, and uncertain blood supply to the bowel edges, a non-restorative approach with the creation of a bowel stoma should be considered [9]. In the described patient's situation, the preferred option was to create a stoma due to the nature of the surgery being damage-controlled, and the potential risk of a leak associated with performing an anastomosis.

The goal of surgical treatment in CD is not curative but rather aimed at resolving complications. Common indications for surgery include disease refractory to medical therapy, uncontrollable bleeding, obstructive strictures, fistulizing disease, bowel perforation, and suspected malignancy [10]. Surgery should be considered earlier in the treatment course for patients with recalcitrant CD, rather than viewing surgery as a failure of medical management [11]. Emergency surgery should be avoided whenever possible due to its associated morbidities and a higher rate of stoma formation. Based on a systematic review and meta-analysis conducted by Linnea Samsø et al., urgent surgery for Crohn's disease is associated with higher overall postoperative complications [12]. In the presented case, despite receiving optimal medical therapy, the patient had isolated ileocaecal CD complicated by complex fistulation of the large and small bowels, localized strictures, and a collection in the right iliac fossa, necessitating urgent surgery. Instead of prolonged exposure to long-term steroid and immunosuppressive therapy, with its associated disease-specific complications, or facing admission and emergency surgery, patients may benefit from early surgical intervention when resistance to medical therapy is evident.

## Conclusions

The optimal treatment approach for patients with CD continues to be a topic of debate, with the primary goal being to enhance quality of life and minimize the physical and psychological impact of the disease. When medical treatment fails to yield the desired results, delays in surgical intervention should be minimized, and patients should be promptly referred to experienced surgeons for appropriate management. It is crucial to emphasize that surgical decisions should be made through a multidisciplinary approach, involving surgical intervention in conjunction with optimal medical and nutritional therapy, to ensure the best possible care for patients with CD.
